# Density-Dependent Prevalence of *Francisella tularensis* in Fluctuating Vole Populations, Northwestern Spain

**DOI:** 10.3201/eid2308.161194

**Published:** 2017-08

**Authors:** Ruth Rodríguez-Pastor, Raquel Escudero, Dolors Vidal, François Mougeot, Beatriz Arroyo, Xavier Lambin, Ave Maria Vila-Coro, Isabel Rodríguez-Moreno, Pedro Anda, Juan J. Luque-Larena

**Affiliations:** Universidad de Valladolid, Palencia, Spain (R. Rodríguez-Pastor, J.J. Luque-Larena);; Instituto Universitario de Investigación en Gestión Forestal Sostenible, Palencia (R. Rodríguez-Pastor, J.J. Luque-Larena);; Instituto de Salud Carlos III, Madrid, Spain (R. Escudero, A.M. Vila-Coro, I. Rodríguez-Moreno, P. Anda);; Universidad de Castilla-La Mancha, Ciudad Real, Spain (D. Vidal);; Instituto de Investigación en Recorsosos Cinégeticos del Consejo Superior de Investigaciones Científicas, Ciudad Real (F. Mougeot, B. Arroyo);; University of Aberdeen, Aberdeen, Scotland, UK (X. Lambin)

**Keywords:** Francisella tularensis, bacteria, tularemia, density-dependent prevalence, prevalence, voles, fluctuating populations, outbreaks, wildlife, zoonoses, Spain

## Abstract

Tularemia in humans in northwestern Spain is associated with increases in vole populations. Prevalence of infection with *Francisella tularensis* in common voles increased to 33% during a vole population fluctuation. This finding confirms that voles are spillover agents for zoonotic outbreaks. Ecologic interactions associated with tularemia prevention should be considered.

Emerging infectious zoonotic diseases are increasing worldwide, and most zoonoses are linked to wildlife (*1**,**2*). Thus, quantifying disease prevalence in potential wildlife hosts is critical to understanding the outbreak dynamics of zoonoses (*3*). Tularemia, which is caused by *Francisella tularensis*, is a problematic zoonotic disease worldwide, but its ecology remains poorly understood. This pathogen is classified by the US Centers for Disease Control and Prevention as a class A bio-threat agent (*F. tularensis* subsp. *holarctica* in Europe) because only a few bacteria are needed to induce tularemia in humans or susceptible animal species (>250 hosts described) (*4*). However, the relative epidemiologic roles (i.e., reservoir, spillover, and amplification agents) for different hosts are uncertain.

A major hotspot for tularemia in Europe is northwestern Spain (Castilla and León region), where the largest recent outbreaks of the disease have been recorded (>1,000 officially confirmed human cases during 1997–1998 and 2007–2008) (*5*). In intensive farmlands in Europe, rodents and lagomorphs are the main putative mammalian hosts (*5*,*6*), but most studies addressing the epidemiologic roles of these species have been correlative or used opportunistic sampling.

A recent study suggests that common voles (*Microtus arvalis*) are a key agent for human tularemia in northwestern Spain because of a spatial and temporal coincidence between human tularemia cases and increases in number of voles (*5*). Voles periodically fluctuate in density and can reach high numbers during specific periods in farming areas (*5*). Dead voles infected with *F. tularensis* subsp. *holarctica* have been reported in northwestern Spain during massive decreases in vole populations (*7*). If, as hypothesized (*5*), common voles are a key amplifying and spillover agent for tularemia in intensive farming areas in northwestern Spain, we should expect an increased prevalence of tularemia in voles as their numbers increase. Thus, it is crucial to empirically evaluate whether such a density-dependent pattern occurs in natural populations.

We obtained samples from live voles periodically collected during population increases (2013–2015) in northwestern Spain. Our goal was to determine how prevalence of *F. tularensis* in common voles varies with population density.

## The Study

We complied with all necessary licenses and permits for conducting this study. During 2013–2015, a common vole population fluctuation, which peaked in 2014, was observed in agricultural areas of Castilla and León, Spain (*8*). This increase in vole numbers was moderate (in terms of peak density) compared with previous increases when tularemia outbreaks among humans were reported (1997–1998 and 2007–2008) by the National Network of Epidemiologic Surveillance of Spain (*5*,*8*). In 2014, no outbreak of tularemia was reported. However, there was a higher-than-average number of identified cases of tularemia (n = 95) among humans in the area (the regional average is 3 [range 0–11] cases/y, excluding outbreak years) (*5*).

To monitor vole abundance during the complete population fluctuation, we sampled 80 km^2^ of farmland in Palencia Province, Spain (42°1′N, 4°42′W), where human tularemia cases have been reported (*5*,*8*). We live-trapped voles seasonally (every 4 months) during March 2013–March 2015. Our vole trapping effort was constant (840 traps set for 24 h/seasonal sampling), and our sampling design was spatially stratified (we obtained random samples from 8 alfalfas fields, 8 grain fields, and 8 fallow fields at each seasonal sampling). Vole abundance was estimated as the number of captures/100 traps/24 h in each season. Trapping was extractive, and animals were brought alive to our laboratory in rodent cages provided with food, water, and bedding immediately after their capture. Voles were euthanized by using CO_2_. Carcasses were individually frozen at −30°C.

We extracted DNA from a homogenized mixture of liver and spleen (≈25 mg). DNA was extracted by using standard procedures (QIAamp DNA Mini Kit; QIAGEN, Valencia, CA, USA). A phylogenetically informative region of the lipoprotein A (*lpnA*) gene (231 bp) was amplified by conventional PCR and hybridized with specific probes by reverse-line blotting as described (*9*). We tested positive samples by using a real-time multitarget TaqMan PCR and tul4 and ISFtu2 assays (*10*). Negative controls for PCR and DNA extraction were included in each group of samples processed. We used R 3.2.4 software (https://stat.ethz.ch/pipermail/r-announce/2016/000597.html) for statistical analyses.

We tested 243 live voles and found an average prevalence of *F. tularensis* of 20.16%. Prevalence greatly varied between samplings (range 0%–33%) and was strongly related to vole abundance (generalized linear model, χ^2^ = 21.64, df = 1, p<0.001) with a direct and positive density-dependent association (Figure). The predicted odds of tularemia infection increased by 1.037 (95% CI 1.021–1.056) when vole density increased by +1 captured vole/100 traps/24 h (range during the study 1–60 voles/100 traps/24 h). During the vole population peak in July 2014, a total of 34 (33%) of 102 sampled live voles were infected with *F. tularensis*.

**Figure:**
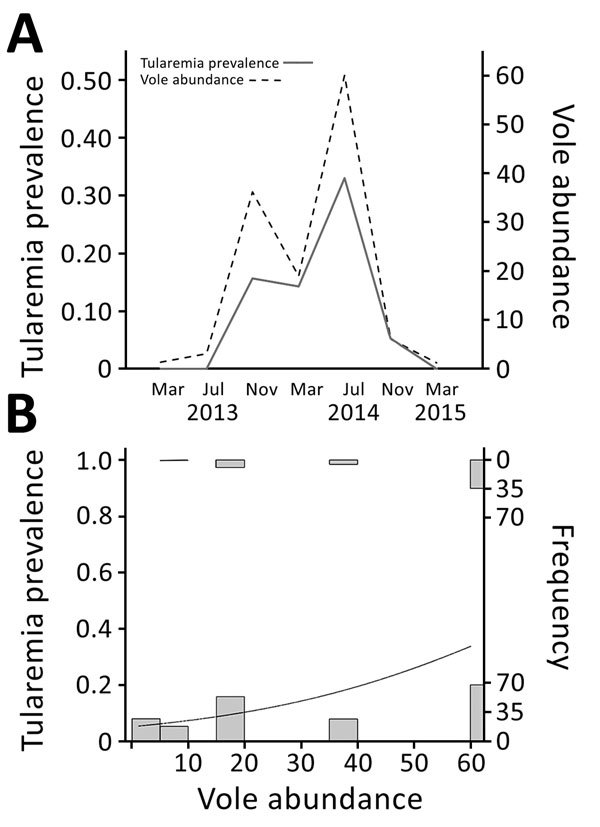
Vole abundance and tularemia prevalence, northwestern Spain. A) Temporal variations in vole abundance (no. captures/100 traps/24 h) and tularemia prevalence. Four voles were tested in March 2013, 15 in July 2013, 32 in November 2013, 63 in March 2014, 102 in July 2014, 19 in November 2014, and 8 in March 2015. B) Relationship between tularemia prevalence and vole abundance. Histograms show number of positive (top) or negative (bottom) voles sampled at each level of vole density. Curved line indicates a generalized linear model result.

## Conclusions

We report a direct and positive density-dependent association between prevalence of *F. tularensis* in common voles and their abundance in agricultural landscapes. These findings are consistent with vole-to-vole transmission and amplification of the bacterium as vole density increases. Voles experimentally infected with *F. tularensis* die within a few days after rapid acute infection and generally show high bacterial loads in organs (*11*). Thus, transmission between voles might involve direct contact, cannibalism, or contamination of the environment. In our study, all voles tested were alive and free of obvious signs of disease when captured, which implied that prevalence could be higher than what we estimated if moribund voles were less trappable and underrepresented in trapped animals.

The role that exogenous sources (i.e., other animals, environmental sources) might play in modulating infection prevalence among vole populations still needs to be clarified. Notwithstanding and irrespective of the precise mechanism(s) of transmission, our results support the hypothesis that exponential growth of common vole populations is crucial for amplification of tularemia transmission in farmlands, and that increases in vole populations are linked to periodic emergence of human cases of tularemia in Spain (*5*). Vole density can reach >1,000 voles/hectare (i.e., >300 tularemia-infected voles/hectare) during outbreaks, potentially leading to contamination of the environment and other wildlife, including harvestable species, such as crayfish and hares, which have higher contact rates with humans than voles (*5*).

Tularemia is probably not completely enzootic in vole populations because we did not detect *F. tularensis* at low densities of voles, which suggests involvement of animal or environmental reservoirs. A key unknown facet of the ecologic cycle of *F. tularensis* is where does it persist between epizootic periods (*5*,*6*). There is no evidence for *F. tularensis* replication in arthropods. However, ticks might be a reservoir of this pathogen because they have life-long infections. Thus, mammalian populations are probably needed to amplify tularemia in the environment (*11*).

Characteristic spatial and social behaviors of voles, including increased contact rates, aggression, and wounding, during massive population increases readily account for amplification of disease transmission rates and spread (*5*,*12*). Although reservoir and vector hosts of *F. tularensis* at variable densities can play major roles in the ecologic cycle of tularemia in different ecosystems, there appears to be a common link between tularemia outbreaks and rodent population fluctuations across Europe (*5*,*6*,*11*,*13*).

Common voles are useful for surveillance of tularemia, and strategic prevention programs should incorporate their temporal fluctuations in planned preventive actions. Because vole numbers seem to modulate the risk for disease exposure in humans, monitoring vole population dynamics can help anticipate and increase awareness of the risk for tularemia in rural areas of Spain.
